# Comparative evaluation of non-surgical and surgical treatment modalities for oral cancer

**DOI:** 10.6026/973206300221374

**Published:** 2026-03-31

**Authors:** Akhil Trivedi, JK Savita, Mayuri Jain, Devashree Shukla, Monika Tanwar, Mona Janak Shah, Tanvi Hirani

**Affiliations:** 1 Department of Dentistry, Government Medical College, Orai, Jalaun, Uttar Pradesh, India; 2 Department of Oral Pathology & Microbiology, Rajarajeswari Dental College & Hospital, Bangalore, India; 3 Department of Oral Medicine and Radiology, Maharana Pratap College of Dentistry and Research Centre, Gwalior, Madhya Pradesh, India; 4 Department of Dentistry, LN Medical College and J K Hospital, Kolar Road, Bhopal, Madhya Pradesh, India; 5 Department of Oral and Maxillofacial Surgery, SGT University, Gurgaon, Haryana, India; 6 Department of Oral Medicine & Radiology, Dharmsinh Desai University, Nadiad, Gujarat, India; 7 Department of Periodontology and Implantology, Karnavati School of Dentistry, Karnavati University, Gandhinagar, Gujarat, India

**Keywords:** Oral cancer, oral squamous cell carcinoma (OSCC), surgical treatment, chemoradiotherapy, survival outcomes, treatment effectiveness

## Abstract

Oral cancer treatment decisions between surgery and non-surgical approaches critically impact survival and quality of life outcomes worldwide. This retrospective cohort study compared surgical treatment (n=98) versus definitive chemoradiotherapy (n=82) in 180 patients with oral squamous cell carcinoma diagnosed 2017-2021. The surgical group demonstrated superior 3-year overall survival (68.4% vs 52.4%, p=0.018) and disease-free survival (61.2% vs 44.0%, p=0.012). Locoregional recurrence was significantly lower with surgery (24.5% vs 39.0%, p=0.032), while distant metastasis rates remained comparable (14.3% vs 17.1%, p=0.608). Surgery provides superior oncologic outcomes for early, localised oral squamous cell carcinoma, while non-surgical options remain valuable for inoperable cases or significant comorbidities.

## Background:

Oral cancer is a significant health issue among the population around the globe and oral squamous cell carcinoma (OSCC) makes up about 90 per cent of all oral cavity-related malignancies [1]. Oral cancer is found worldwide, where the number of incidences and deaths exceeds 375 and nearly 180,000 cases/year, respectively. Interestingly, South and Southeast Asian nations such as India are characterised by disproportionately high incidence rates due to high tobacco chewing and betel quid use and alcohol consumption [2]. Oral cancer therapy has been changing significantly over the past few decades; nowadays, the treatment plans are multimodal, involving surgical, radiotherapy, chemotherapy and more targeted immunotherapeutic drugs and agents [3]. The choice of treatment option is dependent on a wide range of issues such as: tumour stage, anatomical location, patient performance status, comorbidities, functional status and patient preferences. With therapeutic inventions, the five-year oral cancer survival is not optimal and has a range of 50-65 per cent at all stages of the disease, with more advanced disease showing a significantly worse prognosis [4]. Oral cancer treatment is still based on surgical resection with reasonable margins that give conclusive pathological staging data and the best local disease control [5]. Modern surgical practice includes wide local excision, composite resection including mandibulectomy or maxillectomy and complete neck dissection to manage the lymph nodes in the region. Developments in reconstructive measures, such as the free transfer of microvascular free tissues, have made a significant contribution towards functional and aesthetic rehabilitation in ablative surgery [6]. Non-surgical treatment modalities, which are mainly comprised of definitive chemoradiotherapy, have the potential of organ preservation and can be used as alternatives to patients experiencing unresectable lesions or some serious surgical contraindications [7]. Simultaneous delivery of platinum-based chemotherapy and radiotherapy is superior to radiotherapy alone about loco regulation and survival and has become the treatment of choice in the management of locally advanced disease as a non-surgical intervention. The further advancements of therapeutic ratios are the ability of intensity-modulated radiotherapy to allow dose escalation and reduce the toxicity on normal tissues [8]. The comparative performance of non-surgical and surgical interventions is a field of further research and a subject of clinical controversy. Direct comparisons of the modalities are ethically and logistically difficult and this makes the randomised controlled trials very difficult in the determination of comparative effectiveness [9]. The current literature has provided inconsistent outcomes depending on the selection of patients, nature of the disease, treatment regimens and experience of the institution. Several retrospective studies have proposed better survival after primary surgery methods, especially in early-stage oral tongue cancer and locally advanced disease that lends itself to surgery [10]. The results of other studies have, however, shown similar effects between modalities where suitable treatment is accordingly matched with disease characteristics and patient factors. The oral cancer symptoms are heterogeneous and the protocols used to manage the disease are also different, with complications in that the reporting of the outcome is also not the same [11]. The issue of quality of life complicates the choice of treatment. Surgical resection can lead to severe functional loss in areas such as speech, swallowing and appearance, although through reconstructive advances, these issues are minimal [12]. On the other hand, chemoradiotherapy causes acute and late toxicities such as mucositis, xerostomia, dysphagia and osteoradionecrosis, which affect the long-term functional and quality of life. The comparative functional effectiveness of treatment modalities is an aspect that must be evaluated with oncological performance. Although the literature on the treatment outcomes of oral cancers is extensive, there are still gaps in available research on comparative effectiveness in a clinical context involving real-life cases, especially among populations that have a high oral cancer burden. The characteristics of Indian patient cohorts are distinct, such as high stages of presentation, different risk factors and unique anatomical patterns, which may respond to the treatment differently than the Western cohorts [13]. Therefore, it is of interest to compare the efficacy of surgical versus non-surgical treatment of patients with oral squamous cell carcinoma (overall survival, disease-free survival, locoregional recurrence, distant metastasis and treatment-related complications).

## Materials and Methods:

## Study design and setting:

This retrospective cohort study was conducted at a tertiary care oncology centre in India, analysing patients treated between January 2017 and December 2021.

## Inclusion criteria:

[1] Histopathologically confirmed oral squamous cell carcinoma

[2] Primary tumour sites including oral tongue, floor of mouth, buccal mucosa, gingiva, hard palate, retromolar trigone and lip

[3] Age ≥18 years at diagnosis

[4] Clinical stage I-IVA (AJCC 8th edition staging)

[5] Eastern Cooperative Oncology Group (ECOG) performance status 0-2

[6] Complete treatment received as per institutional protocols

[7] Minimum follow-up of 12 months or death within the follow-up period

[8] Complete medical records available for analysis

## Exclusion criteria:

[1] Oropharyngeal, hypopharyngeal or laryngeal primary tumours

[2] Distant metastasis at presentation (Stage IVB/IVC)

[3] Previous treatment for head and neck malignancy

[4] Concurrent second primary malignancy

[5] Palliative treatment intent

[6] Incomplete treatment due to patient non-compliance

[7] Lost to follow-up within 12 months without a documented outcome

## Treatment protocols:

## Surgical group:

The patients have been subjected to primary surgery resection with curative intent. Surgical interventions were used to do wide local excision with 1-1.5 cm clinical margins, partial or total glossectomy, marginal or segmental mandibulectomy, maxillectomy and floor of mouth excisions, depending upon the extent of the tumour. Selective, modified radical or radical neck dissection according to nodal staging was done. Depending on the nature of the defects, reconstruction was done using primary closure, local flaps, regional pedicled flaps, or microvascular free tissue transfer. The adjuvant therapy was performed according to the risk factors of the pathology. The use of adjuvant radiotherapy (60-66 Gy in 30 -33 fractions) was suggested in case of pT3-4 tumours, positive lymph nodes, perineural invasion, or lymphovascular invasion. Positive or close surgical margins and extranodal extension were treated with adjuvant concurrent chemoradiotherapy comprising a regimen of weekly cisplatin (40 mg/m ^2^).

## Non-surgical group:

There was definitive chemoradiotherapy of external beam radiotherapy (70 Gy) in 35 fractions in 7 weeks with concurrent chemotherapy based on platinum. The chemotherapy included cisplatin weekly (40 mg/m^2^) or three weeks of cisplatin (100 mg/m^2^) on day 1, 22 and 43. The patients who were contraindicated to cisplatin were given either weekly carboplatin (AUC 1.5-2) or cetuximab. When it was available, intensity-modulated radiotherapy was used. The choice of treatment was based on multidisciplinary tumour board assessment in relation to the tumour resectability, expected proportion of functional performance, patient performance condition, comorbidities and the patient's preferences.

## Data collection:

The data were obtained based on medical records that comprised demographic (age, gender), risk factor (tobacco use, alcohol use, betel quid use), clinical presentation, tumour (anatomical site, clinical stage, histological grade), treatment, pathology (in surgical patients), treatment complications, recurrence and survival.

## Outcome measures:

## Primary outcomes:

[1] Overall survival (OS): This is the time between treatment start and death by any cause or final follow-up.

[2] Disease-free survival (DFS): Time since coming out of treatment to first recurrence (locoregional or distant) or death.

## Secondary outcomes:

[1] Locoregional recurring rate.

[2] Distant metastasis rate

[3] Complications associated with treatment.

[4] Functional outcome (subjective evaluation of speech and swallowing)

## Follow-up protocol:

The patients received the follow-up procedures in accordance with the institutional guidelines: after 6-8 weeks in the first year, after 3 months in the second year, after 4-6 months in the third-fifth years and at least annually in the sixth-eighth years. Follow-Up assessments were to consist of clinical assessment, imaging (CT or MRI) at 3 months follow-up and, as clinically warranted, chest radiography on a yearly basis.

## Statistical analysis:

The statistical analysis was done in SPSS 26.0 (IBM Corporation) and R 4.1.0. Continuous variables were provided in Mean±SD or median (interquartile range) and the categorical variables were in forms of frequencies and percentages. Between-group comparisons made use of the independent samples t-test or Mann-Whitney U test in continuous variables and chi-square test or Fisher's exact test in categorical variables. The analysis used survival analysis based on a Kaplan-Meier approach using the log-rank test to compare the results between the groups. Cox proportional hazards regression was used to determine independent variables that were predictive of survival with hazard ratios (HR) and 95% confidence interval (CI). To solve selection bias, propensity score matching was conducted, which matched on the basis of age, stage, performance status and the comorbidity index. The p-value of less than 0.05 was found to be statistically significant.

## Results:

The mean age of participants was 54.2 ± 11.8 years, with no significant difference between the surgical (53.4 ± 11.2 years) and non-surgical groups (55.2 ± 12.4 years) (p = 0.298). Age group distribution was also comparable between groups (p = 0.342). The majority of patients were male (76.1%) and gender distribution did not differ significantly between the two groups (p = 0.186). Lifestyle risk factors such as tobacco use (84.4%), alcohol consumption (37.8%), and betel quid use (53.3%) were common in the study population, with no statistically significant differences between surgical and non-surgical groups (p > 0.05). However, significant differences were observed in some clinical parameters. A higher proportion of patients in the surgical group had better ECOG performance status (0-1: 85.7%) compared with the non-surgical group (70.7%), which was statistically significant (p = 0.042). The Charlson Comorbidity Index was significantly higher in the non-surgical group (2.8 ± 2.0) than in the surgical group (2.1 ± 1.6) (p = 0.012), indicating greater comorbidity burden among non-surgical patients. Regarding tumor characteristics, clinical T stage (p = 0.048) and overall stage (p = 0.034) showed significant differences, with early-stage tumors (T1-T2 and Stage I-II) being more common in the surgical group, while advanced disease was more frequent in the non-surgical group. In contrast, clinical N stage, primary tumor site, and histological grade showed no significant differences between groups (p > 0.05) (Table 1 - see PDF). Regarding overall survival (OS), the surgical group showed better outcomes compared with the non-surgical group. The 1-year OS was 89.8% in the surgical group and 82.9% in the non-surgical group, though this difference was not statistically significant (p = 0.172). However, 2-year OS (78.6% vs 65.9%) and 3-year OS (68.4% vs 52.4%) were significantly higher in the surgical group (p = 0.048 and p = 0.018, respectively). Similarly, disease-free survival (DFS) was better among surgically treated patients. The 1-year DFS was 82.7% in the surgical group and 72.0% in the non-surgical group, though this difference was not significant (p = 0.082). However, 2-year DFS (71.4% vs 54.9%) and 3-year DFS (61.2% vs 44.0%) were significantly higher in the surgical group (p = 0.018 and p = 0.012). The median DFS was also longer in the surgical group (48.6 months) compared with the non-surgical group (26.8 months) (p = 0.012). In terms of recurrence patterns, overall recurrence occurred in 32.7% of surgical patients and 46.3% of non- surgical patients, which approached statistical significance (p = 0.058). Locoregional recurrence was significantly higher in the non- surgical group (39.0%) compared with the surgical group (24.5%) (p = 0.032). Specifically, local recurrence alone was significantly more common in the non-surgical group (26.8%) than in the surgical group (14.3%) (p = 0.036). However, regional recurrence, combined local and regional recurrence, and distant metastasis showed no significant differences between groups. The mean time to recurrence was significantly longer in the surgical group (14.8 ± 8.6 months) compared with the non-surgical group (11.2 ± 6.4 months) (p = 0.042). Regarding mortality, total deaths were significantly higher in the non-surgical group (53.7%) than in the surgical group (36.7%) (p = 0.022). Similarly, disease-related deaths were more frequent in the non-surgical group (46.3%) compared with the surgical group (30.6%) (p = 0.028). In contrast, treatment-related deaths and deaths from other causes did not differ significantly between groups (Table 2 - see PDF). Overall, acute complications were significantly higher in the non-surgical group (75.6%) compared with the surgical group (42.9%) (p<0.001). Severe mucositis (58.5% vs 16.3%), dermatitis (26.8% vs 8.2%), dysphagia requiring feeding tube (46.3% vs 22.4%), neutropenia (22.0% vs 6.1%), and nephrotoxicity (9.8% vs 2.0%) were significantly more common among non-surgical patients. In the surgical group, procedure-related complications included surgical site infection (18.4%), wound dehiscence (12.2%), flap failure (6.1%), and hemorrhage requiring intervention (4.1%). Similarly, late complications were significantly higher in the non-surgical group (63.4%) than in the surgical group (38.8%) (p = 0.001). The non-surgical group had markedly higher rates of xerostomia (70.7% vs 18.4%), trismus (29.3% vs 8.2%), osteoradionecrosis (14.6% vs 4.1%), persistent dysphagia (34.1% vs 16.3%), hypothyroidism (17.1% vs 6.1%), and fibrosis (39.0% vs 12.2%), all showing statistically significant differences. However, lymphedema did not differ significantly between the groups (p = 0.136). Regarding functional outcomes, speech impairment was similar in both groups (22.4% vs 22.0%, p = 0.938). However, moderate-to-severe swallowing impairment was significantly higher in the non-surgical group (41.5%) compared with the surgical group (24.5%) (p = 0.014). Feeding tube dependence at one year and return to oral diet did not show statistically significant differences, although feeding tube dependence was higher in the non-surgical group (14.6% vs 6.1%) (Table 3 - see PDF).

## Discussion:

This retrospective cohort study has shown that surgical intervention is better than non-surgical intervention as a way of treating oral squamous cell carcinoma in patients. There was a much better overall survival, disease-free survival and reduced locoregional recurrence in the surgical group in 3 years. The findings speak in favour of the further relevance of surgery as the first-line treatment modality in resectable cases of oral cancer and in favour of the usefulness of the non-surgical options in patient groups. The observed survival benefit using surgical treatment is consistent with the large retrospective series and database studies on the outcome of oral cancer treatment. All population-based studies that have been conducted using the National Cancer Database have reported better survival using primary surgical treatment in oral cavity squamous cell carcinoma at various stages [14]. The intrinsic benefits of surgery are full pathological evaluation to focus on proper selection of adjuvant therapy, eradication of possible hypoxic areas of tumor which might be radioresistant and immediate elimination of the disease instead of gradual regression of the tumour. The much smaller locoregional recurrence rate of the surgical group (24.5% versus 39.0) is explained by the more effective local control that was attained by full surgical excision with satisfactory margins. Locoregional failure is the most common mode of treatment failure in oral cancer and has a great effect on survival. The recurrence patterns studied have always found the local failure as the most likely location of first- time treatment failure after the chemoradiotherapy [15]. The fact that surgery has the capability of providing negative margins, which is proved by the use of intraoperative frozen section in cases where it is required, gives a higher confidence of a full removal of the tumour. The reduced survival benefit in Stage IVA disease (50.0 versus 47.5) indicates that may be that advanced locoregional disease may surpass the difference in therapeutic benefits between therapeutic modalities. This observation agrees with the fact that the choice of treatment modality is less likely to predict outcomes the deeper the disease progresses, with tumour biology taking the leading role [16]. Nevertheless, even similar outcomes of survival can be in support of surgery where the treatment-related morbidity in the properly chosen patients is low. The increased rate of acute complications in the non-surgical group (75.6% versus 42.9%) is mainly caused by the mucosal and systemic toxicities of concurrent chemoradiotherapy. Severe mucokinetis is the prevalent type of mucokinetis (58.5% of the patients of chemoradiotherapy), which results in a lot of pain, loss of nutritional functions, loss of treatment and poor quality of life [17]. Although surgical complications such as infection and wound dehiscence have clinical implications, they are time-bound and most frequently correctable compared to the cumulative and, in most cases, progressive radiotherapy-induced toxicities. Late complications showed even greater differences between groups, with xerostomia, trismus, osteoradionecrosis and fibrosis showing significant differences after chemoradiotherapy. These delayed effects are the irreversible effects that have significant effects on the quality of life in the long-term [18]. Xerostomia, which occurs in 70.7 per cent of the patients receiving chemoradiotherapy as compared to 18.4 per cent of the patients who undergo surgery with adjuvant therapy, brings about chronic discomfort, dental caries and problems in swallowing that progress over time. The similarity in the rates of speech impairment of both groups (22.4 versus 22.0) hints at the possibility of the claims about the surgical morbidity being partially negated by the radiation-impaired fibrosis and neural damage of articulation. Contemporary re-creative procedures, especially microvascular free tissue transfer, allow functional replacement using even ablative procedures [19]. The fact that the impairment of swallowing was, in fact, more significant in the non-surgical group (41.5% versus 24.5) contradicts the assumptions that the preservation of the organs automatically leads to the preservation of the functions. The selection bias of the treatment choice that is present in a retrospective comparison is one of the factors that should be taken into account when interpreting these findings. The patients who are to receive definitive chemoradiotherapy are usually those who miss disease features or comorbidities, which make them poor surgical candidates. This selection pattern is verified by our discovery that the non-surgical group had significantly higher Charlson Comorbidity Index and worse performance status. To some degree, propensity score matching dealt with this bias and the preserved survival advantage in matched analysis enhanced causal inference [20]. The use of adjuvant therapy after surgery is a role that should be considered in comparative effectiveness assessment. Sixty-three per cent of the patients who underwent surgery were given adjuvant therapy; that is, bimodal and trimodal therapy. The comparison is therefore between surgery plus adjuvant therapy and definitive chemoradiotherapy as opposed to surgery alone and chemoradiotherapy. This is an indicator of the practice patterns in the real world, where adjuvant therapy is usually recommended depending on the pathological results [21]. The institutional experience and number of patients probably contribute to the results of both treatment options. High-volume centres have a high level of performance with complex surgical operations and maximised supportive care in the management of chemoradiotherapy toxicity. These results may not be applicable in smaller volume facilities where there is less experience in both forms of treatment [22]. Whether a surgical or non-surgical procedure should be used depends on a case-by-case evaluation, including oncological, individual, functional and access to the treatment as key factors. Tumour board evaluation is multidisciplinary, which means it is through in its treatment planning. The decision-making processes that involve shared decision-making ought to offer patients realistic outcome expectations of both modalities and thus make informed decisions that suit their values [23]. New treatment models, such as the incorporation of immunotherapy and de-escalation techniques, can change the relative effectiveness field. Immune checkpoint inhibitors are currently researched in the neoadjuvant, adjuvant and definitive settings. New approaches like response-adapted approaches with the use of induction chemotherapy or immunotherapy to select patients to preserve the organ are promising directions that could allow a more personal approach to the distribution of treatment [24]. There are various limitations with this study. The retrospective design presents intrinsic bias in spite of the propensity score manipulation. The single-institution information can restrict generality. The determination of quality of life was based on functional outcomes and not patient-reported outcome measures that have been validated. The treatment procedures changed through the course of the study, bringing in time heterogeneity. Also, the follow-up can be rather short, thus underestimating the occurrence of lateness and long-term complications.

## Conclusion:

We show that surgical management is superior to chemoradiotherapy for oral squamous cell carcinoma, achieving higher 3-year overall survival and disease-free survival, particularly in early/intermediate stages, including Stage IVA. Surgical treatment showed lower locoregional recurrence with better functional swallowing despite chemoradiotherapy's higher acute toxicity and late xerostomia/trismus/ fibrosis complications. Primary surgery optimises outcomes for resectable disease while chemoradiotherapy serves unresectable cases; multidisciplinary assessment incorporating patient comorbidities, functional priorities and preferences should guide treatment selection.
